# Antibiotic Use Before CAR-T Treatment Is Associated with Inferior Outcomes in DLBCL Lymphoma Patients

**DOI:** 10.3390/biomedicines14020298

**Published:** 2026-01-29

**Authors:** Anja Bullegas, Inna Shaforostova, Katja Seipel, Marie-Noëlle Kronig, Henning Nilius, Ulrike Bacher, Thomas Pabst

**Affiliations:** 1 Department of Medical Oncology, Inselspital, University Hospital Bern, University of Bern, 3010 Bern, Switzerland; anja.bullegas@students.unibe.ch (A.B.); innaivanovna.shaforostova@insel.ch (I.S.); katja.seipel@insel.ch (K.S.);; 2 Department for Biomedical Research, University of Bern, 3012 Bern, Switzerland; 3 Center for Laboratory Medicine, Department of Clinical Chemistry, Inselspital, University Hospital Bern, University of Bern, 3010 Bern, Switzerland; henning.nilius@insel.ch; 4 Central Hematology Laboratory, Department of Hematology, Inselspital, University Hospital Bern, University of Bern, 3010 Bern, Switzerland; veraulrike.bacher@insel.ch

**Keywords:** CAR-T-cell therapy, DLBCL, microbiome, antibiotic treatment

## Abstract

**Background/Objectives:** CAR-T-cell therapy has become a key treatment for relapsed or refractory hematologic malignancies such as diffuse large B-cell lymphoma (r/r DLBCL), although patient outcomes differ considerably. The intestinal microbiome has been proposed as an important factor influencing CAR-T-cell therapy efficacy; accordingly, antibiotic exposure, which may induce dysbiosis, has been associated with inferior outcomes after CAR-T-cell therapy. **Methods:** We retrospectively analyzed clinical data from 140 patients to assess the impact of infection-related antibiotic therapy prior to CAR-T-cell therapy, stratifying them into two cohorts: 67 patients with previous antibiotic exposure and 73 without exposure. **Results:** Patients exposed to antibiotics prior to CAR-T therapy had significantly reduced progression-free survival (*p* = 0.016) and overall survival (*p* = 0.002) compared to those without exposure. Multiple antibiotic courses and shorter intervals between the last antibiotic treatment and CAR-T-cell therapy were linked to poorer outcomes. **Conclusions:** Our data suggest that pre-CAR-T-cell-therapy antibiotic exposure is associated with inferior outcomes, although it remains unclear whether this effect is causal or reflects underlying patient comorbidities. These findings highlight the need for further studies investigating the role of antibiotic-induced dysbiosis on CAR-T-cell therapy efficacy.

## 1. Introduction

CAR-T-cell therapy has revolutionized the treatment for refractory malignant hematological diseases such as diffuse large B-cell lymphoma (DLBCL) [1,2,3,4]. Despite initially encouraging response rates, only up to 40% of patients achieve a complete and durable remission [1,2,4,5]. In addition, CAR-T-cell therapy is associated with characteristic adverse events like cytokine release syndrome (CRS) and immune effector cell-associated neurotoxicity syndrome (ICANS) [3,5]. The variability in therapeutic responses, together with the potential for severe toxicities, underscores the need to identify and understand factors that influence the outcome after CAR-T-cell therapy.

One factor that has gained increasing attention in this context is the intestinal microbiome possibly exerting a crucial role in shaping and stimulating the host immune system, making it a key candidate for modulating response rates to immunotherapies such as CAR-T-cell therapy [6,7,8]. It produces a broad spectrum of metabolites known to enhance T-cell function and affect systemic immune activity [1,7,8,9,10,11]. Importantly, the efficacy and sustained persistence of CAR-T-cells are closely associated with clinical outcome, with higher persistence correlating with improved response in patients with B-cell lymphoma [12]. At the same time, CAR-T-cell efficacy and persistence also depend on the intrinsic functionality of T-cells and their stimulation, highlighting the critical role of proper T-cell activation for achieving effective and durable CAR-T-cell responses [4,8,10]. Recent evidence demonstrates that the intestinal microbiome can modulate therapeutic responses and the occurrence of treatment-related complications by affecting immune cell infiltration and inflammatory mediator release within the tumor microenvironment [6,9,13]. Given these findings, the interplay between the intestinal microbiome and the efficacy of CAR-T-cell therapy has become a rapidly evolving field of research, explored in both preclinical and clinical studies. Considering its central role in regulating essential physiological and immunological processes, it is not surprising that microbial composition and function have emerged as critical determinants of CAR-T-cell therapy outcomes [14,15].

Since the intestinal microbiome appears to influence CAR-T-cell responses, factors that disrupt its function are of particular interest. Among these, antibiotic therapy represents one of the most profound and clinically relevant modulators of microbial composition. When administered prior to CAR-T-cell treatment, antibiotics can markedly reduce the high microbial diversity of the intestinal microbiome, a feature considered essential for maintaining physiological function. The resulting dysbiosis may persist for weeks to several months, depending on the type of antibiotic and the duration of exposure [5,16]. In particular, exposure to broad-spectrum antibiotics, including meropenem, piperacillin-tazobactam, and cefepime, has been associated with shorter progression-free survival (PFS) and overall survival (OS), as reported in the study by Smith et al. [5,15].

Given the evidence that antibiotic-induced disruption of the intestinal microbiome may influence CAR-T-cell efficacy and patient outcomes, we conducted a single-center retrospective study to assess whether exposure to antibiotics prior to CAR-T-cell therapy affects clinical response and survival.

## 2. Materials and Methods

### 2.1. Patient Selection

Included in the analyses were all consecutive patients who received CAR-T-cell therapy for relapsed/refractory diffuse large B-cell lymphoma at the University Hospital of Bern, Switzerland, between January 2018 and April 2025. Patients were stratified into two cohorts according to whether they had received antibiotic therapy within six months preceding CAR-T-cell treatment or not. Based on this stratification, cohort A included patients without antibiotic therapy and cohort B included patients who had received antibiotic therapy.

### 2.2. Procedures

Patient data were collected retrospectively. To assess the comparability of the two cohorts, various patient characteristics were recorded, including age at diagnosis, time from diagnosis to CAR-T-cell therapy, IPI Score, prior lines of therapy, ECOG performance status at the time of CAR-T-cell therapy, and comorbidities. For simplification, conditions categorized as cardiovascular or gastrointestinal diseases were grouped accordingly. Cardiovascular diseases included arterial hypertension, coronary heart disease, infarctions (both cerebral and myocardial), cardiac arrhythmias, heart failure, and thrombosis. Gastrointestinal diseases encompassed inflammatory conditions, functional disorders, irritable bowel disease, and chronic infections such as Helicobacter pylori.

Antibiotic therapy due to infection within 6 months prior to CAR-T-cell therapy was recorded from patients’ medical histories and was observed in 67 of 140 patients. To further evaluate the potential impact of antibiotic exposure, an internal stratification of the antibiotic-treated cohort was performed. Two variables were of primary interest: the number of antibiotic courses administered prior to CAR-T-cell therapy and the interval between the last antibiotic exposure and CAR-T-cell infusion. For the purposes of this analysis, each initiated antibiotic regimen was considered an independent therapy, irrespective of subsequent antibiotic changes within the same infectious episode.

For the analysis of antibiotic burden, patients were categorized into two groups: those who received a single antibiotic course and those who received more than one course. In addition, patients were stratified according to the time interval between their last antibiotic administration and CAR-T-cell therapy: 0–7 days and >7 days.

All patients received infection prophylaxis with trimethoprim-sulfametoxazole, fluconazole, and valaciclovir at the time of CAR-T-cell therapy. As there were no differences between the cohorts in this regard, prophylaxis was not considered further in the analysis. Patients in this study received the CD19 CAR-T-cell products Tisagenlecleucel (Kymriah^®^, Novartis, Basel, Switzerland), Axicabtagene ciloleucel (Yescarta^®^, Kite Pharma, Inc., Santa Monica, CA, USA), and Lisocabtagene maraleucel (Breyanzi^®^, Bristol Myers Squibb, Berkeley Heights, NJ, USA), which were distributed approximately equally across both cohorts.

### 2.3. Study Design

This retrospective cohort study evaluated the impact of antibiotic therapy prior to CAR-T-cell therapy on patient outcomes. The primary endpoint was the comparison of progression-free survival (PFS) and overall survival (OS) between the two cohorts over a 24-month observation period. During this period, follow-up data were collected at 3, 6, 12, and 24 months after CAR-T-cell retransfusion.

### 2.4. Statistics

Progression-free survival (PFS) was defined as the time from the day of CAR-T-cell retransfusion until the first relapse, death, or last follow-up. Overall survival (OS) had the same starting point as PFS, and the ending point was death of any cause or last follow-up. Patient data up to 31 October 2025 were included in the evaluation.

GraphPad Prism^®^ version 10.6.1 was used to evaluate the patient data and to visualize the statistics in Kaplan–Meier curves. A multivariate analysis was performed to assess the impact of antibiotic therapy on patient outcomes while adjusting for potential confounding variables such as age, sex, ECOG performance status, number of comorbidities, and remission status prior to CAR-T-cell therapy.

Continuous variables were compared using the *t*-test or the Mann–Whitney U test, depending on whether the data were normally distributed, whereas categorical variables were analyzed using the Chi-square test or Fisher’s exact test, as appropriate.

A *p*-value ≤ 0.05 was considered statistically significant.

## 3. Results

This study included data from 140 DLBCL patients. Of these, 73 had no antibiotic exposure in the six months preceding CAR-T-cell therapy, while 67 had received antibiotic treatment at least once for various indications.

### 3.1. Patient Characteristics

Various baseline characteristics were assessed to evaluate cohort comparability. Overall, the distribution of major variables—including sex, age at diagnosis, IPI Score, and number of comorbidities—was largely similar. Although the total comorbidity burden was comparable, the types differed; for instance, gastrointestinal conditions were more frequent in the antibiotic-treated cohort (Table 1). The antibiotics classes used are shown in Table 2.

Regarding remission status before CAR-T infusion, progressive disease predominated in both groups. However, a higher proportion of patients in the antibiotic-treated cohort presented with progressive disease. A difference was also observed in ECOG performance status at the time of CAR-T-cell retransfusion, with the cohort receiving antibiotic therapy showing a slightly higher average score. The distribution of CAR-T-cell products was comparable between the two cohorts (Table 3).

### 3.2. Adverse Events

Statistically, there was no significant difference between the two cohorts in the incidence of cytokine release syndrome (CRS), immune effector cell-associated neurotoxicity syndrome (ICANS) or hypogammaglobulinemia. However, there was a trend toward higher rates of both CRS and ICANS in the cohort receiving antibiotic therapy. Tocilizumab and steroids were used to a similar extent in both cohorts to manage the adverse events (Table 4).

### 3.3. Clinical Outcome

**Figure 1:**
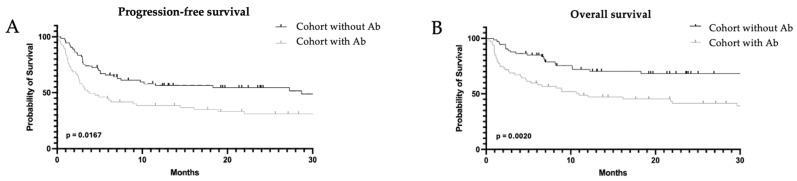
Kaplan–Meier curves illustrating (**A**) progression-free survival of cohort without vs. with antibiotics (Ab) in the last 6 months before CAR-T-cell infusion; (**B**) overall survival of cohort without vs. with Ab.

Clinical outcomes were assessed at 3, 6, 12, and 24 months after CAR-T-cell retransfusion. The best remission status achieved within the 24-month observation period was evaluated alongside length of hospitalization, relapse events, and mortality. Progression-free survival (PFS) and overall survival (OS) were estimated using Kaplan–Meier curves (Figure 1).

The duration of hospitalization was longer in the antibiotic-treated cohort (*p* = 0.04), and a greater proportion of these patients required transfer to the intensive care unit (*p* = 0.06).

Remission status differed significantly between the cohorts: complete remission was more frequently observed in patients who did not receive antibiotics (58% vs. 36%; *p* = 0.03), whereas progressive disease was more frequent in the antibiotic-treated cohort (27% vs. 14%; *p* = 0.09) (Table 5). CR rates did not differ between subgroups with AB intervals of 0–7 days and >7 days (*p* = 0.63). Overall, 56 patients relapsed during the 24-month follow up, with similar proportions across both cohorts; however, the median time to relapse was shorter among antibiotic-treated patients (2 vs. 3 months, *p* = 0.33). A total of 65 patients died, with significantly higher mortality in the antibiotic cohort (61% vs. 33%; *p* = 0.004). Deaths due to disease progression were also more common in the cohort with antibiotic therapy and also the interval between CAR-T infusion and death was significantly shorter (342 vs. 237 days; *p* = 0.002). Both PFS and OS differed significantly in favor of the cohort without antibiotic therapy (PFS, *p* = 0.016; OS, *p* = 0.002), as illustrated by the Kaplan–Meier curves (Figure 1). The observation above was supported by the multivariate analyses for PFS and OS, which demonstrated that the use of antibiotics emerged as an independent adverse prognostic factor (PFS, *p* = 0.023, OS, *p* = 0.003) (Table 6 and Table 7).

### 3.4. Comparison Within the Antibiotic Group

To assess additional factors associated with antibiotic exposure, both the number of antibiotic courses administered and the interval between the last antibiotic intake and CAR-T-cell therapy were analyzed.

**Figure 2:**
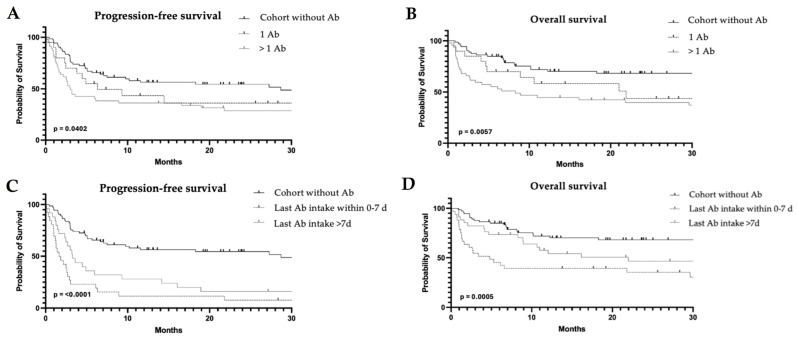
Kaplan–Meier curves illustrating (**A**) progression-free survival between cohort without antibiotics (Ab) vs. 1 Ab vs. >1 Ab, (**B**) overall survival between cohort without Ab vs. 1 Ab vs. >1 Ab, (**C**) progression-free survival between cohort without Ab vs. last intake within 0–7 d vs. >7 d, (**D**) overall survival between cohort without Ab vs. last intake within 0–7 d vs. >7 d.

Patients who received only one antibiotic course demonstrated a median PFS that was twice as long as that of patients who received multiple antibiotic courses (6.3 vs. 3 months; *p* = 0.004). Median OS also differed significantly, with 22 months in the single-antibiotic cohort compared with 6 months in the cohort receiving more than one antibiotic course (*p* = 0.002) (Figure 2A,B).

Significant differences were also observed between the groups stratified by the interval from the last antibiotic intake to CAR-T infusion. Patients with an interval of 0–7 days from the last antibiotic exposure exhibited a median PFS of 1.8 months compared with 3.2 months in patients whose last antibiotic intake occurred more than 7 days prior to CAR-T therapy (*p* = 0.26). Median OS differed significantly between the two groups, with 4.8 months in the 0–7-day cohort vs. 21.9 months in the 7-day cohort (*p* = 0.001) (Figure 2C,D).

## 4. Discussion

In this retrospective cohort study, we investigated whether infection-related antibiotic exposure prior to CAR-T-cell therapy influenced clinical outcomes. Prophylactic antibiotics were not considered, as they were uniformly administered and thus could not serve to separate cohorts. Our analysis showed that patients who received antibiotics prior to CAR-T-cell therapy had significantly worse outcomes, as measured by progression-free survival (*p* = 0.016) and overall survival (*p* = 0.002) within the 24 month observation period. Both the number of antibiotics and the interval between the last dose and CAR-T-cell therapy were associated with inferior outcomes, with patients receiving more than one antibiotic or whose last dose was administered within 0–7 days prior to therapy showing poorer results. However, these associations were less pronounced than the overall effect of any prior antibiotic exposure. Patients with prior antibiotic exposure also experienced longer hospital stays and higher frequency of intensive care unit transfers following CAR-T-cell therapy. Whether this reflects a direct effect of antibiotics or simply indicates a more vulnerable patient population cannot be determined from our data. Notably, a better response was observed in the cohort without prior antibiotics not only in terms of PFS and OS, but also regarding the best remission status within 24 months. The rate of progressive disease shows a trend toward being higher in the cohort with prior antibiotics (*p* = 0.09), whereas the rate of complete remission is significantly higher in the cohort without antibiotics (*p* = 0.03). These observations may indicate that the intestinal microbiome, through its stimulatory effects on immune cells, could play a key role in determining response to CAR-T-cell therapy and that antibiotic-induced dysbiosis may represent a critical negative factor [17,18,19].

These findings are in line with previous reports, which have shown that pre-treatment antibiotic exposure is associated with a reduced response to CAR-T-cell therapy [1,15,17,20]. Limiting unnecessary exposure to antibiotics may help preserve intestinal microbiome diversity, which could contribute to improved clinical outcomes [6,7]. However, it is important to balance this consideration with the need to manage active infections, which can be life-threatening in this patient population. Studies suggest that antibiotic-induced disruption of the intestinal microbiome, particularly changes in the abundance of specific taxa such as Bifidobacterium, Akkermansia, and Clostridia, may impair CAR-T-cell expansion and immune function, providing a plausible explanation for the observed associations [5,15,16]. Several studies support the hypothesis that the intestinal microbiome influences immune function through the production of specific metabolites, thereby affecting the efficacy of immunotherapies such as CAR-T-cell therapy [18,19]. A diverse and functionally intact microbiome is associated with enhanced T-cell fitness and metabolic support, whereas antibiotic-induced dysbiosis can reduce microbial diversity and function, impairing CAR-T-cell expansion and antitumor activity [8,10]. Clinical observations indicate that alterations in gut microbiome composition may correlate with response rates to CAR-T-cell therapy. Supporting this, in vivo experiments and metabolomic analyses have shown that microbiome-derived metabolites, particularly short-chain fatty acids (SCFAs) such as pentanoate or acetate, can modulate T-cell activation, differentiation, and exhaustion, thereby enhancing the antitumor response of CAR-T-cells [8,11]. Multi-omics approaches combining microbiome and metabolomic data are being used to dissect the complex host–microbe–immune interactions that shape CAR-T-cell therapy responses. These data inform strategies to manipulate the microbiome (e.g., through diet, antibiotics, or fecal microbiota transplantation) and metabolic pathways (e.g., ex vivo metabolic reprogramming of CAR-T-cells) to enhance efficacy and reduce toxicity [21,22,23]. Overall, these findings highlight that alterations in the intestinal microbiome and reduced microbial metabolite availability may weaken CAR-T-cell responses and adversely affect therapy outcomes.

The study provides a meaningful comparison between patients with and without prior antibiotic exposure, owing to the cohort size (*n* = 140) and comprehensive clinical data. Nevertheless, the limitations inherent to the retrospective study design must be acknowledged. Selection bias cannot be entirely excluded, even in light of the significant findings from the multivariable analysis, which identified antibiotic exposure—and, with respect to overall survival, the presence of multiple comorbidities—as the only independent predictors of inferior outcomes in this cohort. Ultimately, it remains unclear whether antibiotic administration directly contributed to poorer outcomes, or whether the patients’ clinical condition—including active infection, increased inflammatory activity, and generally worse health at the time of therapy—necessitated antibiotic use. These patients may have exhibited a poorer response to CAR-T-cell therapy even in the absence of antibiotic treatment. This hypothesis is also supported by findings from Prasad et al., who showed that patients with high tumor burden and systemic inflammation were more likely to receive antibiotics prior to CAR-T-cell therapy and that antibiotic exposure was associated with inferior outcomes [17].

Ultimately, it remains unclear whether antibiotic administration itself contributed to poorer outcomes or whether these outcomes were driven by the patients’ clinical condition at the time of therapy—including active infections, increased inflammatory activity, and overall poorer health—that necessitated antibiotic use.

Future prospective studies are needed to confirm these findings and to further investigate the underlying mechanisms, particularly the role of the intestinal microbiome in modulating response to CAR-T-cell therapy. If these results are validated, interventions such as microbiome modulation or targeted antibiotic management could potentially improve outcomes in this vulnerable patient population. Overall, our results highlight the importance of careful consideration of antibiotic use prior to CAR-T-cell therapy.

## 5. Conclusions

In conclusion, our study indicates that infection-related antibiotic therapy prior to CAR-T-cell therapy is associated with poorer clinical outcomes. These findings highlight the need for careful consideration of antibiotic use in this setting and support further research into strategies, including microbiome modulation, to optimize CAR-T-cell therapy efficacy.

## Figures and Tables

**Table 1:** Clinical characteristics at diagnosis.

Parameter	All Patients *n* = 140	Cohort Without Antibiotics *n* = 73	Cohort with Antibiotics *n* = 67	*p*
Number of patients, *n* (%)	140 (100)	73 (100)	67 (100)	
Female, *n* (%)	63 (45)	31 (42.5)	32 (47.8)	0.63
Male, *n* (%)	77 (55)	42 (57.5)	35 (52.2)	
Age at initial diagnosis, median, years (r) ^5^	62 (21–80)	62.5 (24–80)	62 (21–79)	
De novo DLBCL ^1^, *n* (%)	108 (77.1)	56 (76.7)	52 (77.6)	0.9
Transformed DLBCL, *n* (%)	32 (22.9)	17 (23.3)	15 (22.4)	
IPI Score ^2^, median	3	3	3	
0–1, *n* (%)	3 (2.1)	2 (2.7)	1 (1.5)	0.6
2, *n* (%)	20 (14.3)	13 (17.8)	7 (10.4)	0.27
3, *n* (%)	52 (37.1)	25 (34.2)	27 (40.3)	0.45
4–5, *n* (%)	39 (27.9)	20 (27.4)	19 (28.4)	0.86
Unknown ^3^, *n* (%)	26 (18.6)	13 (17.8)	13 (19.4)	0.64
Comorbidities ^4^				
Number of comorbidities, median	2	2	2	
0, *n* (%)	27 (19.3)	16 (21.9)	11 (16.4)	0.54
1, *n* (%)	33 (23.6)	17 (23.3)	16 (23.9)	0.88
2, *n* (%)	43 (30.7)	20 (27.4)	23 (34.3)	0.53
3, *n* (%)	29 (20.7)	15 (20.5)	14 (20.9)	0.88
4, *n* (%)	8 (5.7)	5 (6.8)	3 (4.5)	0.73
Cardiovascular diseases, *n* (%)	67 (47.9)	36 (49.3)	31 (46.3)	0.84
Diabetes mellitus, *n* (%)	25 (17.9)	15 (20.5)	10 (14.9)	0.55
Obesity, *n* (%)	9 (6.4)	6 (8.2)	3 (4.5)	0.49
Gastrointestinal diseases, *n* (%)	38 (27.1)	14 (19.2)	24 (35.8)	**0.04**
Liver diseases, *n* (%)	18 (12.9)	7 (9.6)	11 (16.4)	0.32
Renal insufficiency, *n* (%)	20 (14.3)	9 (12.3)	11 (16.4)	0.57
History of other malignancy, *n* (%)	22 (15.7)	13 (17.8)	9 (13.4)	0.67
Neurological/psychiatric disorders, *n* (%)	16 (11.4)	9 (12.3)	7 (10.4)	0.78
Previous abdominal surgeries, *n* (%)	22 (15.7)	10 (13.7)	12 (17.9)	0.65
None of the above comorbidities, *n* (%)	27 (19.3)	16 (21.9)	11 (16.4)	0.54

^1^ DLBCL = diffuse large B-cell lymphoma; ^2^ IPI Score = International Prognostic Index; ^3^ IPI Score not assessable due to missing data; ^4^ patients can exhibit several of these comorbidities at the same time; ^5^ r = range.

**Table 2:** Antibiotic classes before CAR-T-cell therapy.

Antibiotic Classes, *n* (%) ^1^	Patients, *n* (%)
Penicilline, *n* (%)	25 (37.3)
Cephalosporine, *n* (%)	54 (80.6)
Carbapeneme, *n* (%)	14 (20.9)
Fluorchinolone, *n* (%)	6 (9.0)
Tetrazykline, *n* (%)	4 (6.0)
Glykopeptide, *n* (%)	18 (26.9)
Lipopeptide, *n* (%)	1 (1.5)
Nitroimidazole, *n* (%)	29 (43.3)
Nitrofurane, *n* (%)	1 (1.5)
Lincosamide, *n* (%)	4 (6.0)

^1^ Most patients received more than one antibiotic class.

**Table 3:** Patient characteristics at CAR-T-cell therapy.

Parameter	All Patients *n* = 140	Cohort Without Antibiotics *n* = 73	Cohort with Antibiotics *n* = 67	*p*
ECOG performance status, median ^1^	1	1	1	
0, *n* (%)	58 (41.4)	35 (47.9)	23 (34.3)	0.12
1, *n* (%)	56 (40)	31 (42.5)	25 (37.3)	0.46
2, *n* (%)	17 (12.1)	6 (8.2)	11 (16.4)	0.17
3, *n* (%)	6 (4.3)	1 (1.4)	5 (7.5)	0.21
4, *n* (%)	3 (2.1)	0	3 (4.5)	0.25
Age at CAR-T therapy, median, years (r) ^6^	66.5 (22–82)	69 (25–81)	66 (22–82)	
Months from diagnosis to CAR-T, median	18.3	24.7	16.1	
Lines of therapy prior to CAR-T therapy				
1, *n* (%)	16 (11.4)	11 (15.1)	5 (7.5)	0.2
2, *n* (%)	53 (37.9)	30 (41.1)	23 (34.3)	0.31
3, *n* (%)	68 (48.6)	32 (43.8)	36 (53.7)	1
4, *n* (%)	3 (2.1)	0	3 (4.5)	0.26
Remission status at CAR-T therapy				
CR ^2^, *n* (%)	4 (2.9)	3 (4.1)	1 (1.5)	0.61
PR ^3^, *n* (%)	35 (25)	21 (28.8)	14 (20.9)	0.31
SD ^4^, *n* (%)	10 (7.1)	5 (6.8)	5 (7.5)	1
PD ^5^, *n* (%)	91 (65)	44 (60.3)	47 (70.1)	0.26
CAR-T product				
Kymriah^®^, *n* (%)	61 (43.6)	29 (39.7)	32 (47.8)	0.4
Yescarta^®^, *n* (%)	73 (52.1)	40 (54.8)	33 (49.3)	0.56
Breyanzi^®^, *n* (%)	6 (4.3)	4 (5.5)	2 (3)	0.68

^1^ ECOG Score = Eastern Cooperative Oncology Group performance status, defined on the day of CAR-T-cell therapy; ^2^ CR = complete remission; ^3^ PR = partial remission; ^4^ SD = stable disease; ^5^ PD = progressive disease; ^6^ r = range.

**Table 4:** Adverse events.

	All Patients *n* = 140	Cohort Without Antibiotics *n* = 73	Cohort with Antibiotics *n* = 67	*p*
CRS ^1^				
No CRS, *n* (%)	30 (21.4)	15 (20.5)	15 (22.4)	0.83
Grade 1, *n* (%)	68 (48.6)	41 (56.2)	27 (40.3)	0.1
Grade 2, *n* (%)	37 (26.4)	16 (21.9)	21 (31.3)	0.17
Grade 3, *n* (%)	4 (2.9)	1 (1.4)	3 (4.5)	0.61
Grade 4, *n* (%)	1 (0.7)	0	1 (1.5)	1
ICANS ^2^				
No ICANS, *n* (%)	92 (65.7)	49 (67.1)	43 (64.2)	0.74
Grade 1, *n* (%)	17 (12.1)	12 (16.4)	5 (7.5)	0.17
Grade 2, *n* (%)	8 (5.7)	2 (2.7)	6 (9)	0.13
Grade 3, *n* (%)	19 (13.6)	9 (12.3)	10 (14.9)	0.78
Grade 4, *n* (%)	4 (2.9)	1 (1.4)	3 (4.5)	0.61
Administration of Tocilizumab, *n* (%)	95 (67.9)	45 (61.6)	50 (74.6)	0.15
Administration of Steroids, *n* (%)	92 (65.7)	47 (64.4)	45 (67.2)	0.79
Hypogammaglobulinemia ^3^, *n* (%)	118 (84.3)	58 (79.5)	60 (89.6)	0.14

^1^ CRS: Cytokine release syndrome; ^2^ ICANS: immune effector cell-associated neurotoxicity syndrome; ^3^ documented within the 24-month observation period following CAR-T-cell therapy.

**Table 5:** Outcomes.

	All Patients *n* = 140	Cohort Without Antibiotics *n* = 73	Cohort with Antibiotics *n* = 67	*p*
Duration of hospitalization, mean, days	23.3	21.1	25.5	**0.04**
ICU ^1^ stay, *n* (%)	22 (15.7)	7 (9.6)	15 (22.4)	0.06
No ICU stay, *n* (%)	118 (84.3)	66 (90.4)	52 (77.6)	0.06
Best response within 24 months ^2^				
Complete remission, whole cohort, *n* (%)	66 (47.1)	42 (57.5)	24 (35.8)	**0.03**
Interval 0–7 days, *n* (%)			10/33 (30.3)	**0.63**
Interval > 7 days, *n* (%)			14/34 (42.4)	
Partial remission, *n* (%)	41 (29.3)	20 (27.4)	21 (31.3)	0.7
Stable disease, *n* (%)	5 (3.6)	1 (1.4)	4 (6)	0.36
Progressive disease, *n* (%)	28 (20)	10 (13.7)	18 (26.9)	0.09
Relapse, *n* (%)	56 (40)	26 (35.6)	30 (44.8)	0.32
Time to relapse ^3^, median months (r) ^8^	3 (0–30)	3 (0–30)	2 (0–18)	0.33
Death, *n* (%)	65 (46.4)	24 (32.9)	41 (61.2)	**0.004**
Deaths due to progression, *n* (%)	35 (25)	12 (16.4)	23 (34.3)	**0.04**
Non-progression-related deaths, *n* (%)	30 (21.4)	12 (16.4)	18 (26.9)	0.24
Time to death ^3^, median days	290	342	237	**0.002**
PFS med ^4^, months	15.9	n.r. ^9^	3.8	**0.016**
PFSR ^5^ 6 mo %	54.2	65.2	43.2	
PFSR 12 mo %	47.4	56.3	38.4	
PFSR 24 mo %	42.8	54.5	31	
OS med ^6^, months	n.r ^9^	n.r ^9^	11	**0.002**
OSR ^7^ 6 mo %	71.6	84.9	58.2	
OSR 12 mo %	59.5	71.9	47.1	
OSR 24 mo %	54.9	68.2	41.5	
Median follow-up, months	20	21.6	18.4	0.05

^1^ ICU = intensive care unit; ^2^ using follow-up data collected at 3, 6, 12, and 24 months after CAR-T-cell therapy; ^3^ since the day of CAR-T-cell therapy; ^4^ PFS med = median progression-free survival; ^5^ PFSR = progression-free survival rate; ^6^ OS med = median overall survival; ^7^ OSR = overall survival rate; ^8^ r = range; ^9^ n.r. = not reported.

**Table 6:** Multivariant analysis for progression-free survival.

	Univariable Regression				Multivariable Regression		
Characteristic	N	HR ^1^	95% CI ^2^	*p*-Value	HR ^1^	95% CI ^2^	*p*-Value
Group	140						
without Ab ^7^		—	—		—	—	
with Ab ^7^		1.74	1.12, 2.69	**0.014**	1.67	1.07, 2.60	**0.023**
Age	140						
<65 y		—	—		—	—	
≥65 y		1.07	0.69, 1.66	0.8	0.95	0.60, 1.50	0.8
Comorbidities	140						
0–1		—	—		—	—	
2 and more		1.38	0.88, 2.16	0.2	1.36	0.86, 2.17	0.2
Remission status	140						
CR ^3^/PR ^4^		—	—		—	—	
SD ^5^/PD ^6^		1.06	0.66, 1.71	0.8	1.21	0.73, 2.02	0.5
CART product used	140						
Kymriah		—	—		—	—	
Yescarta/Breyanzi		0.76	0.49, 1.18	0.2	0.71	0.45, 1.14	0.2

^1^ HR = hazard ratio; ^2^ CI = confidence interval; ^3^ CR = complete remission; ^4^ PR = partial remission; ^5^ SD = stable disease; ^6^ PD = progressive disease; ^7^ Ab = antibiotics.

**Table 7:** Multivariant analysis for overall survival.

	Univariable Regression				Multivariable Regression		
Characteristic	N	HR ^1^	95% CI ^2^	*p*-Value	HR ^1^	95% CI ^2^	*p*-Value
Group	140						
without Ab ^7^		—	—		—	—	
with Ab ^7^		2.26	1.37, 3.74	**0.002**	2.14	1.29, 3.55	**0.003**
Age	140						
<65 y ^8^		—	—		—	—	
≥65 y ^8^		1.62	0.97, 2.69	**0.066**	1.38	0.82, 2.34	0.2
Comorbidities	140						
0–1		—	—		—	—	
2 and more		1.91	1.14, 3.20	**0.014**	1.73	1.01, 2.95	**0.044**
Remission status	140						
CR ^3^/PR ^4^		—	—		—	—	
SD ^5^/PD ^6^		1.20	0.70, 2.07	0.5	1.38	0.78, 2.46	0.3
CART product used	140						
Kymriah		—	—		—	—	
Yescarta/Breyanzi		0.72	0.44, 1.17	0.2	0.65	0.38, 1.09	0.10

^1^ HR = hazard ratio; ^2^ CI = confidence interval; ^3^ CR = complete remission; ^4^ PR = partial remission; ^5^ SD = stable disease; ^6^ PD = progressive disease; ^7^ Ab = antibiotics; ^8^ y = years.

## Data Availability

The data presented in this study are available upon request from the corresponding author.
